# Supplementary material for: mTOR-related synaptic pathology causes autism spectrum disorder-associated functional hyperconnectivity
Source: Nat Commun. 2021 Oct 19;12:6084. doi: 10.1038/s41467-021-26131-z (PMC8526836; doi:10.1038/s41467-021-26131-z)
Supplement: Supplementary file 1 — Supplementary Information [file 41467_2021_26131_MOESM1_ESM.pdf]

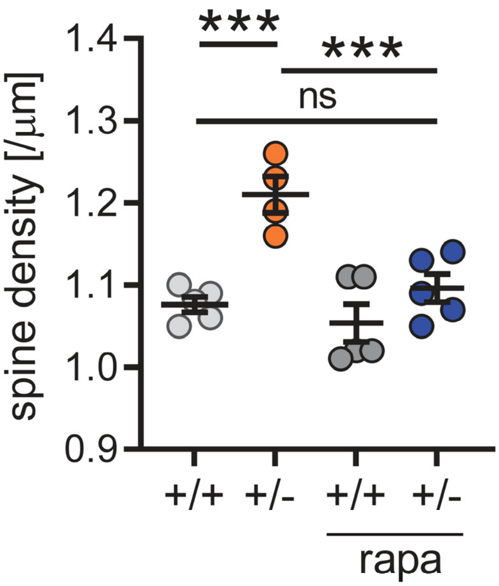

Supplementary Figure S1. Increased dendritic spine density in the retrosplenial cortex of *Tsc2*$^{+/-}$ mice is rescued by rapamycin. In keeping with our prior measurements, we found increased spine density in the retrosplenial cortex of *Tsc2*$^{+/-}$ mice (orange circles, $q < 0.001$, FDR-corrected). This alteration was completely rescued by pharmacological treatment with rapamycin (blue circles, $q < 0.001$, FDR-corrected). $n = 5$ (+/+), $n = 4$ (+/-), $n = 5$ (+/+ rapa), $n = 5$ (+/- rapa). Error bars represent SEM. Source data are provided as a Source Data file.

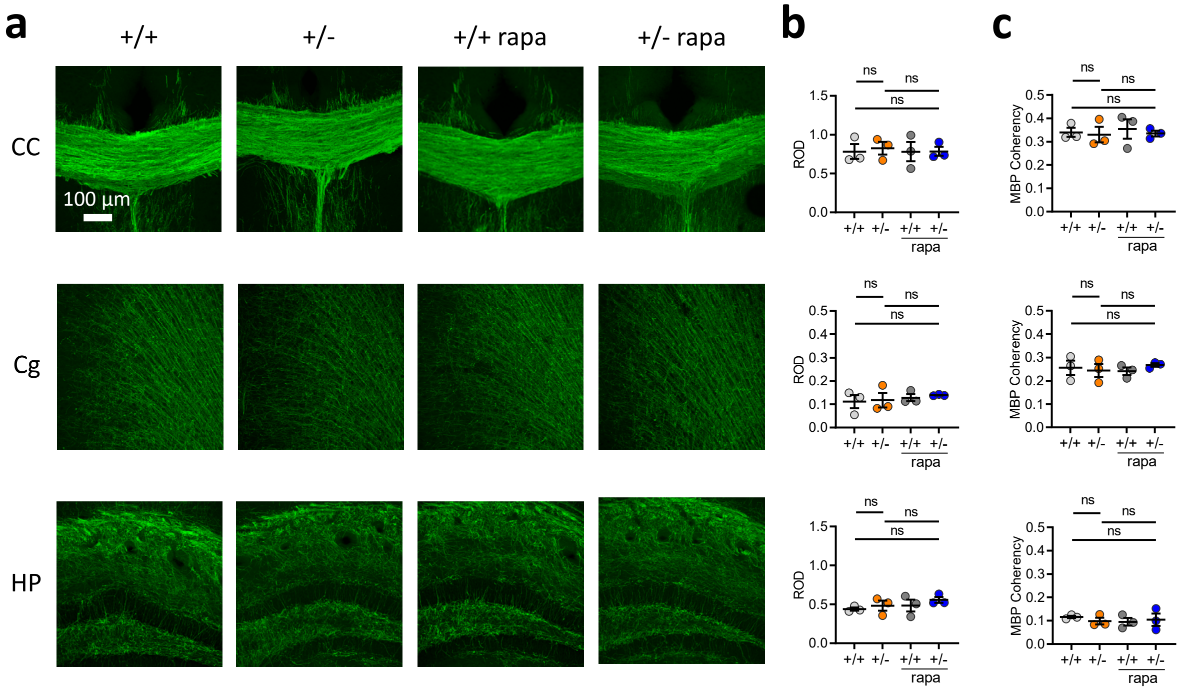

Supplementary Figure S2. Myelin basic protein staining. a) Representative sections showing myelin basic protein (MBP) staining in different brain regions. Scale bar = 100 µm b) Myelin basic protein densitometry in the corpus callosum (CC, top panel), cingulate cortex (Cg, middle panel) and hippocampus (HPC, bottom panel) in control and *Tsc2*$^{+/-}$ mice treated with vehicle or rapamycin (rapa). c) MBP coherency in CC, Cg, and HPC in control or *Tsc2*$^{+/-}$ mice treated with vehicle or rapamycin. ROD: relative optical density value. Panel (b) - (c). *n* = 3 mice per group. Unpaired t-test, two-sided, FDR-corrected. Error bars represent SEM. Source data are provided as a Source Data file.

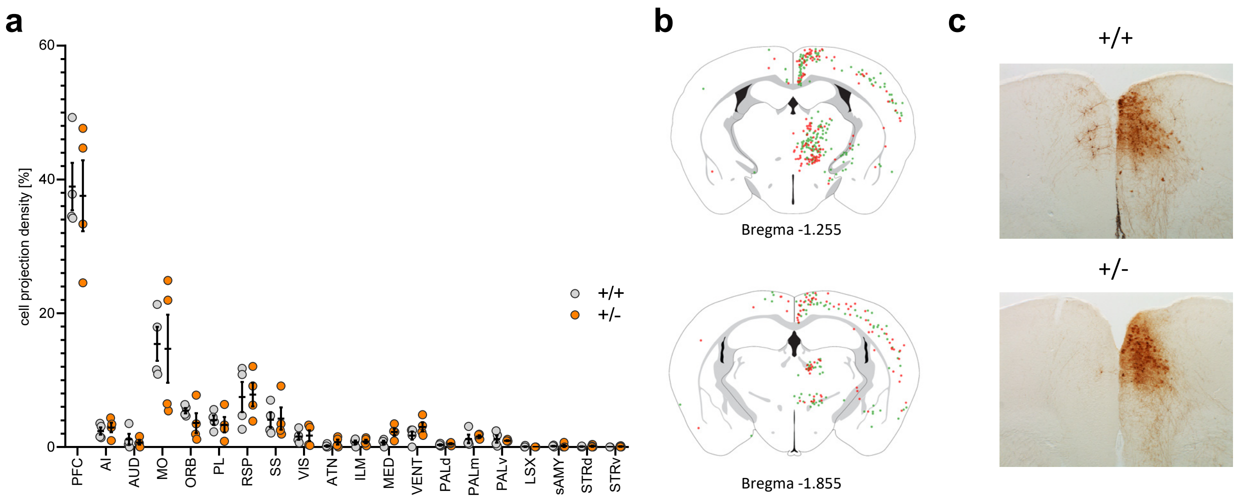

Supplementary Figure S3. Recombinant rabies virus tracing of cell projections to PFC. a) Regional quantifications revealed unimpaired cell projection density in *Tsc2*$^{+/-}$ mice vs. control littermates (q > 0.76, FDR-corrected, all regions). To avoid viral batch effects and operator-dependent biases our measurements have been normalized for total number of marked cells as in (Bertero et al., 2018; Pagani et al., 2019). b) Distribution of cells projecting to PFC as mapped with viral tracing in representative coronal slices (top panel, Bregma = -1.255, and bottom panel, Bregma = -1.855) in *Tsc2*$^{+/-}$ mice (green dots) and control mice (red dots). c) Extension of site of infection in representative control (top panel) and *Tsc2*$^{+/-}$ (bottom panel) mice. n = 4 mice per group. Error bars represent SEM. Source data are provided as a Source Data file.

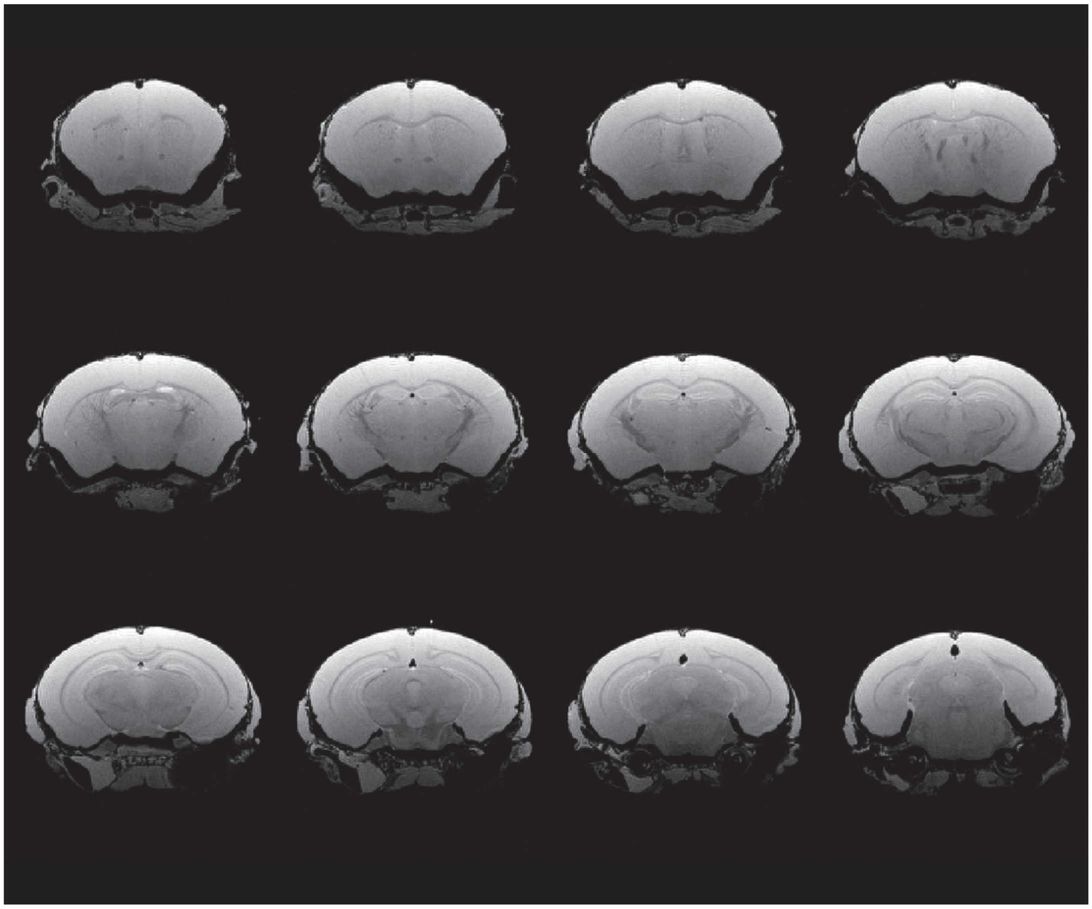

Supplementary Figure S4. High-resolution morpho-anatomical imaging confirmed the absence of tubers in *Tsc2*$^{+/-}$ mice. The figure depicts a set of coronal cuts from a representative *Tsc2*$^{+/-}$ mouse brain.

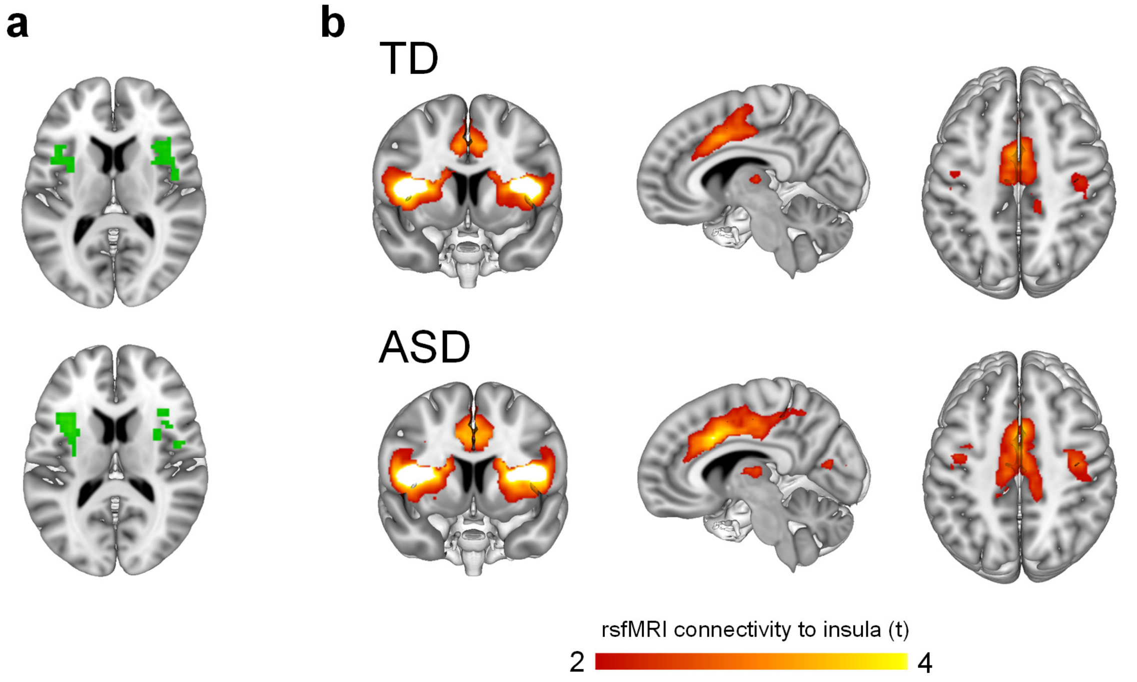

Supplementary Figure S5. Functional connectivity of insular cortex as measured with seed-based mapping.
a) Seed location (in green). b) Seed based network of insular cortex in TDs and individuals with ASD.

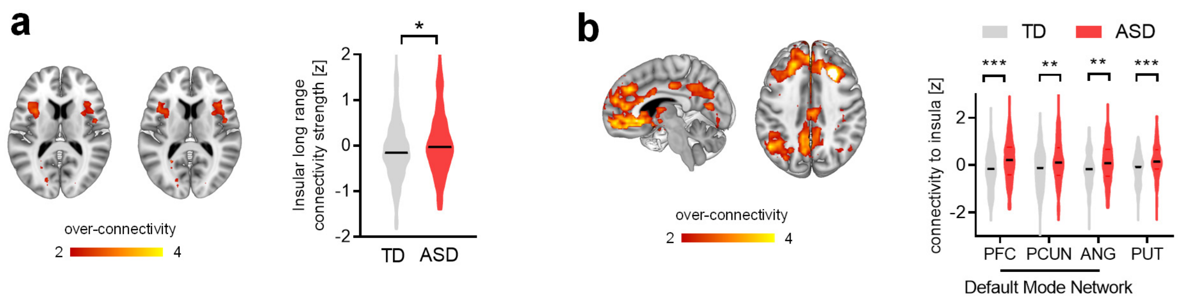

Supplementary Figure S6. Insular hyperconnectivity in children with ASD is not driven by a single collecting site. a) By excluding all scans acquired at Stanford University (i.e. the participating institution with the most prominent case-control hyperconnectivity [Cohen's *d* = 0.81]), long-range rsfMRI connectivity mapping confirmed the presence of bilateral functional connectivity of anterior insular cortices in children with ASD vs TDs (unpaired t-test, two-sided, $t = 2.39$, $p = 0.017$). b) Similarly, seed-based mapping on the same scans corroborated long-range hyperconnectivity between insular and cortico-striatal targets, suggesting that long range hyperconnectivity in ASD is not primarily attributable to time-series acquired at Stanford University. Regional quantification of rsfMRI connectivity between insula and PFC (unpaired t-test, two-sided, $t = 3.91$, $p = 0.001$), precuneus (unpaired t-test, two-sided, $t = 3.22$, $p = 0.002$), angular gyrus (unpaired t-test, two-sided, $t = 3.09$, $p = 0.002$) and putamen (unpaired t-test, two-sided, $t = 4.30$, $p = 0.001$) in ASD vs. TDs. PFC, prefrontal cortex; PCUN, precuneus; ANG, angular gyrus; PUT, putamen. $^*p < 0.05$, $^{**}p < 0.01$, $^{***}p < 0.001$. Error bars represent SEM. Source data are provided as a Source Data file.

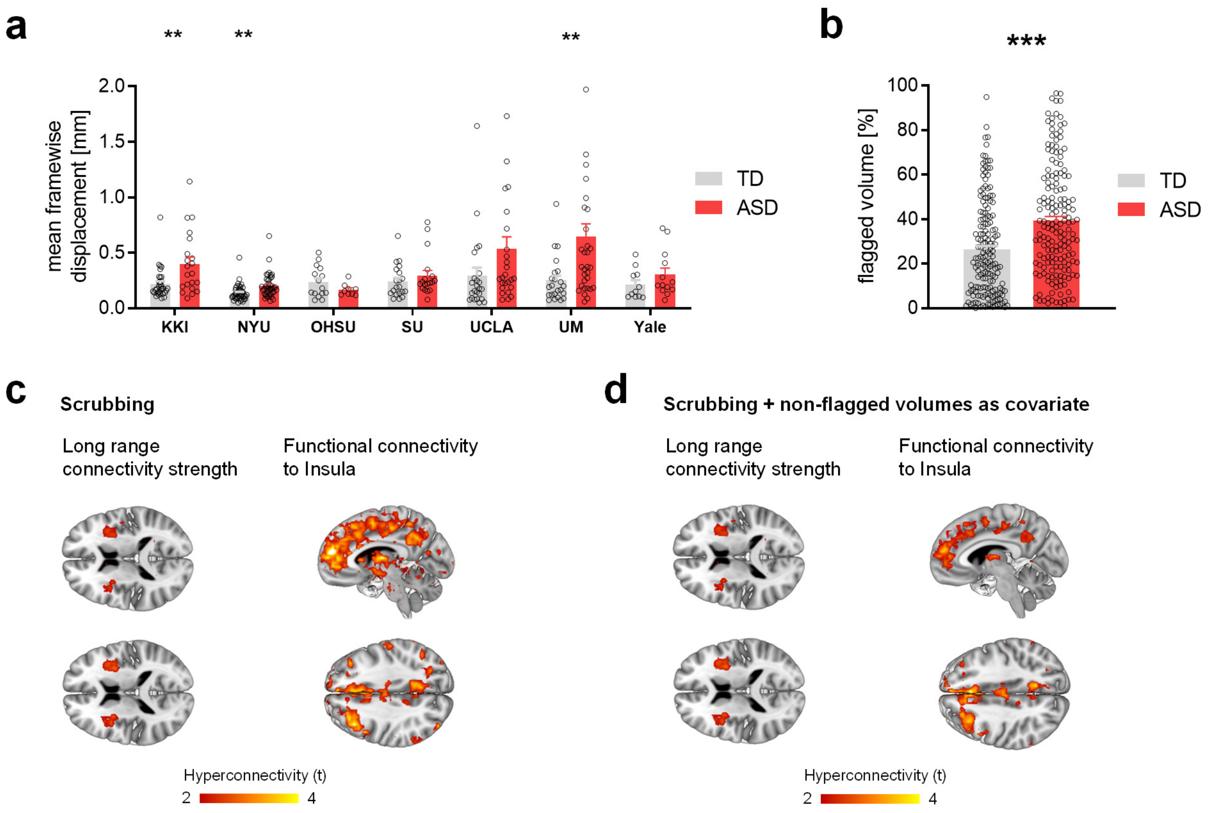

Supplementary Figure S7. Insular hyper-connectivity in children with ASD is identifiable also after strict control of in-scanner head motion with data scrubbing. a) In-scanner head motion during fMRI acquisition as measured with mean frame-wise displacement. Mean frame-wise displacement (FD) is higher in brain scans of children with ASD acquired at KKI (unpaired t-test, two-sided, $t = 3.21$, $p = 0.002$), NYU (unpaired t-test, two-sided, $t = 3.29$, $p = 0.001$) and UM (unpaired t-test, two-sided, $t = 2.86$, $p = 0.006$) as compared to site-matched TDs. b) Percentage of volumes flagged with FD > 0.2 mm (t-test, two-sided, $t = 5.21$, $p < 0.001$). $n = 163$ (ASD), $n = 168$ (TD) for panel (a) and (b). c) Upon removal of volumes showing FD > 0.2 mm, we retained only time-series with at least 50% of the original number of volumes (Tang et al., 2020) for further functional connectivity analysis. Due to the low sample size (ASD, $n = 9$ and CTR, $n = 15$) and the limited number of time-points (volumes, $n = 82$) of the OHSU site, subjects scanned at OHSU were removed from subsequent analyses. In total, for this confirmatory analysis we retained $n = 108$ individuals with ASD and $n = 128$ CTR subjects. Long-range connectivity mapping confirmed bilateral hyperconnectivity in anterior insular regions in children with ASD. Seed based mapping carried out on the same scrubbed data corroborated long-range hyperconnectivity between anterior insular areas and cortico-striatal targets. d) As length of the time-series of ASD subjects (number of volumes, mean = 133, sd = 45, range = 58-289) was shorter than that of CTR subjects (number of volumes, mean = 157, sd = 52, range = 58-294), we also included the number of volumes as a nuisance covariate in our statistical testing of functional connectivity. This set of analysis once again confirmed insular functional hyper-connectivity in the ASD cohort. $**p <$

0.01, ***$p < 0.001$. Error bars in panels (a) and (b) represent SEM. Source data are provided as a Source Data file.

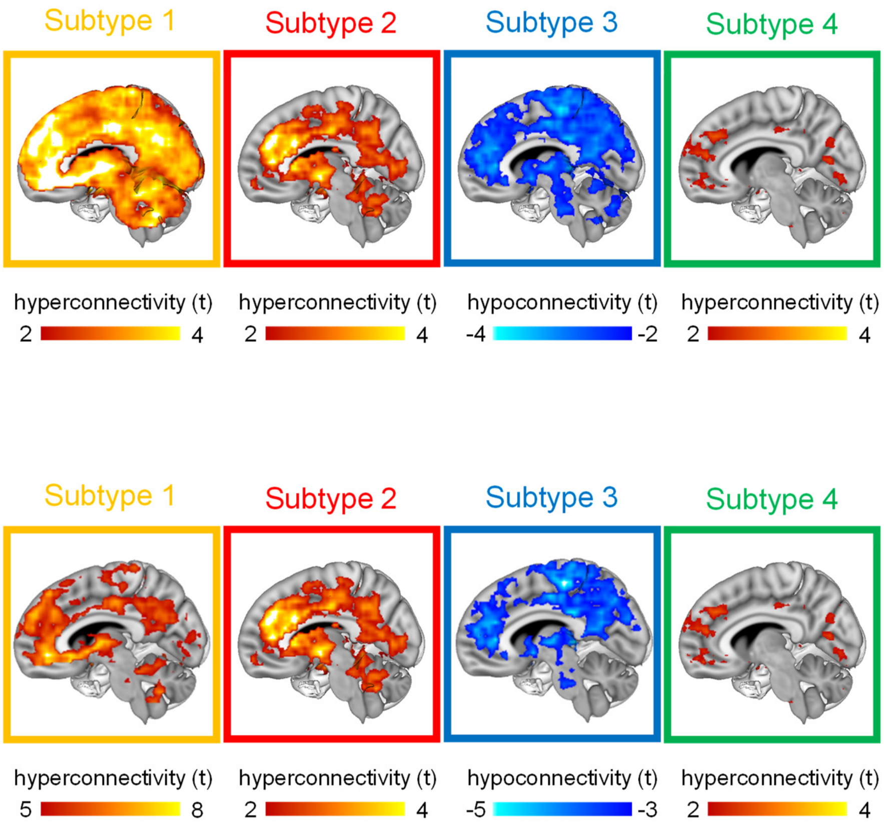

Supplementary Figure S8. Subtype dependent regional specificity of insular hyper- and hypo-connectivity in ASD. Regional specificity of target regions of hyperconnectivity in subtype 1 and hypoconnectivity of subtype 3 is not apparent when a single t-threshold is used across clusters (top, Figure 5c), reflecting different effect size for the different subtypes. Subtype-specific connectional features are however apparent when an arbitrary subtype-dependent threshold is applied to the less spatially undifferentiated subtype 1 ($|t| > 5$, FWER cluster corrected) and subtype 3 ($|t| > 3$, FWER cluster corrected) (bottom).

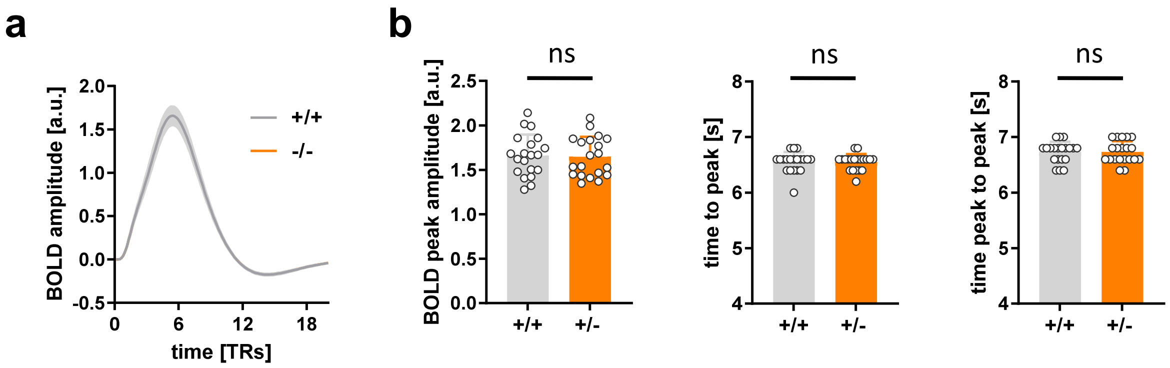

Supplementary Figure S9. Hemodynamic response function exhibits canonical features in *Tsc2*$^{+/-}$ mice. a) Hemodynamic response in PFC in *Tsc2*$^{+/-}$ mutants and +/+ control mice. Error bands represent SEM. b) BOLD peak amplitude, time to peak and FWHM of the hemodynamic response in *Tsc2*$^{+/-}$ mice are comparable to the corresponding parameters observed in control mice (unpaired t-test, two-sided). *n* = 20 per group. Error bars represent SEM. Source data are provided as a Source Data file.

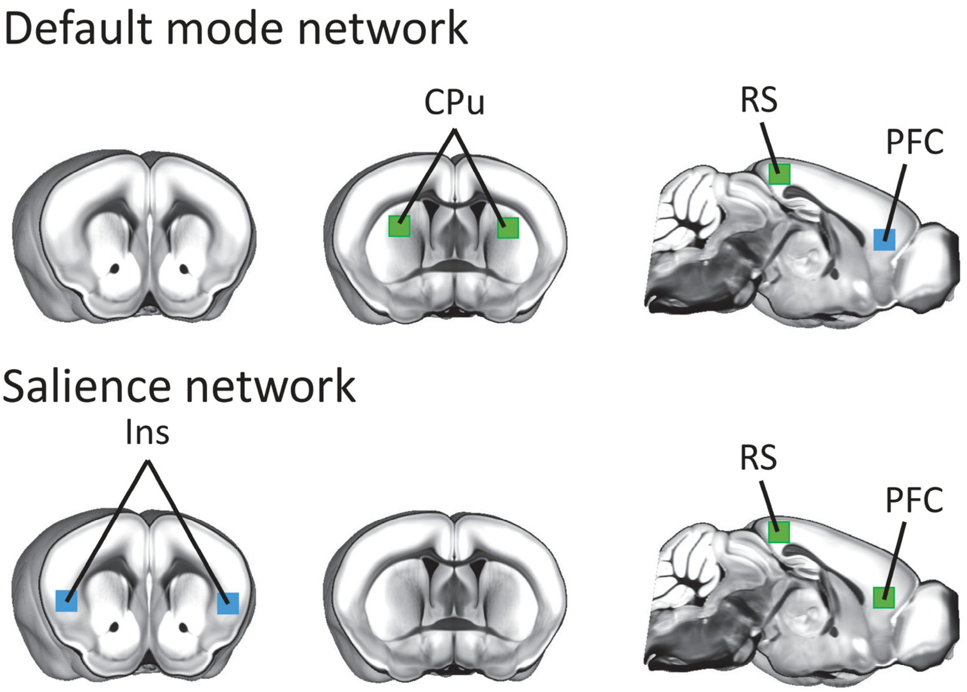

Supplementary Figure S10. Anatomical location of seed regions and volumes of interest. Seed based functional connectivity was quantified by computing correlation between the seed (3*3*1 voxels, in blue) and network-specific volumes of interests (3*3*1 voxels, in green).

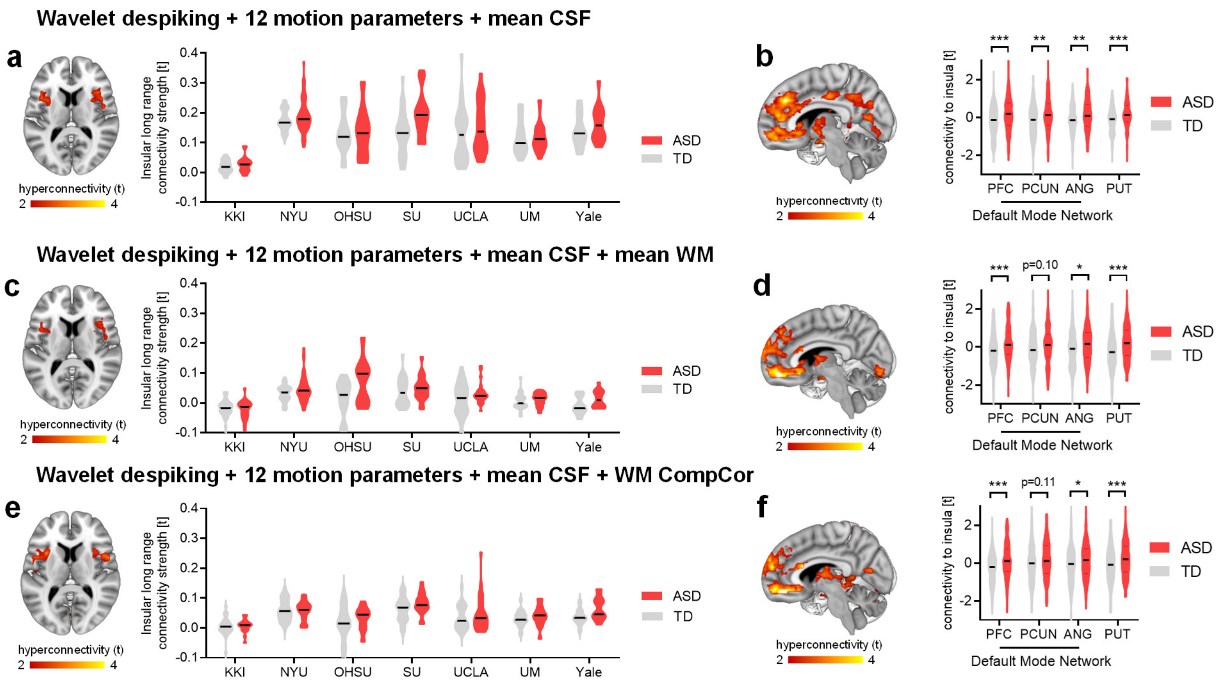

Supplementary Figure S11. rsfMRI connectivity mapping upon mean white matter and white matter CompCorr regression. (a) Global connectivity mapping and (b) seed based correlation analysis carried out following the original denoising pipeline of our work, i.e. wavelet despiking, 12 motion parameters and mean CSF regression as in Patel et al., 2014. Regional quantifications of rsfMRI connectivity between insula and PFC (unpaired t-test, two-sided, t = 3.94, p = 0.001), precuneus (unpaired t-test, two-sided, t = 3.26, p = 0.002), angular gyrus (unpaired t-test, two-sided, t = 3.13, p = 0.002) and putamen (unpaired t-test, two-sided, t = 4.33, p = 0.001) in ASD vs. TD. These are the same results we report in Figure 4. (c) Global connectivity mapping and (d) seed-based correlation analysis carried out following addition of mean white matter regression. Regional quantifications of rsfMRI connectivity between insula and PFC (unpaired t-test, two-sided, t = 3.80, p < 0.001), precuneus (unpaired t-test, two-sided, t = 1.64, p = 0.10), angular gyrus (unpaired t-test, two-sided, t = 2.49, p = 0.013) and putamen (unpaired t-test, two-sided, t = 4.02, p < 0.001) in ASD vs. TD. (e) Global connectivity mapping and (f) seed based correlation analysis carried out following regressions of principal components of the white matter signal as implemented in ANTsR (https://www.rdocumentation.org/packages/ANTsR/versions/1.0). Regional quantifications of rsfMRI connectivity between insula and PFC (unpaired t-test, two-sided, t = 4.52, p < 0.001), precuneus (unpaired t-test, two-sided, t = 1.57, p = 0.11), angular gyrus (unpaired t-test, two-sided, t = 2.32, p = 0.020) and putamen (unpaired t-test, two-sided, t = 2.71, p = 0.007) in ASD vs. TD. All the denoising strategies produced similar foci of hyper-connectivity in insular cortices (a-c-e) and fronto-insulo-striatal hyper-connectivity in ASD scans (b-d-e) arguing against a spurious origin for the hyper-connectivity we mapped in children with ASD. *p < 0.05, **p < 0.01, ***p < 0.001. Error bars represent SEM. Source data are provided as a Source Data file.
