# Supplementary material for: mTOR-related synaptic pathology causes autism spectrum disorder-associated functional hyperconnectivity
Source: Nat Commun. 2021 Oct 19;12:6084. doi: 10.1038/s41467-021-26131-z (PMC8526836; doi:10.1038/s41467-021-26131-z)
Supplement: Supplementary file 2 — Reporting Summary [file 41467_2021_26131_MOESM2_ESM.pdf]

nature research

Corresponding author(s): Alessandro Gozzi

Last updated by author(s): 03/09/2021

# Reporting Summary

Nature Research wishes to improve the reproducibility of the work that we publish. This form provides structure for consistency and transparency in reporting. For further information on Nature Research policies, see our Editorial Policies and the Editorial Policy Checklist.

## Statistics

For all statistical analyses, confirm that the following items are present in the figure legend, table legend, main text, or Methods section.

| n/a | Confirmed | |
|---|---|---|
| ☐ | ☒ | The exact sample size ($n$) for each experimental group/condition, given as a discrete number and unit of measurement |
| ☐ | ☒ | A statement on whether measurements were taken from distinct samples or whether the same sample was measured repeatedly |
| ☐ | ☒ | The statistical test(s) used AND whether they are one- or two-sided<br>*Only common tests should be described solely by name; describe more complex techniques in the Methods section.* |
| ☐ | ☒ | A description of all covariates tested |
| ☐ | ☒ | A description of any assumptions or corrections, such as tests of normality and adjustment for multiple comparisons |
| ☐ | ☒ | A full description of the statistical parameters including central tendency (e.g. means) or other basic estimates (e.g. regression coefficient) AND variation (e.g. standard deviation) or associated estimates of uncertainty (e.g. confidence intervals) |
| ☐ | ☒ | For null hypothesis testing, the test statistic (e.g. $F$, $t$, $r$) with confidence intervals, effect sizes, degrees of freedom and $P$ value noted<br>*Give P values as exact values whenever suitable.* |
| ☒ | ☐ | For Bayesian analysis, information on the choice of priors and Markov chain Monte Carlo settings |
| ☐ | ☒ | For hierarchical and complex designs, identification of the appropriate level for tests and full reporting of outcomes |
| ☐ | ☒ | Estimates of effect sizes (e.g. Cohen's $d$, Pearson's $r$), indicating how they were calculated |

*Our web collection on statistics for biologists contains articles on many of the points above.*

## Software and code

Policy information about availability of computer code

| | |
|---|---|
| Data collection | Mouse MRI scans were acquired with 7T Bruker Biospin with Bruker Paravision software (v6). Images of dendritic spines and MBP immunostaining were acquired with a Nikon A1 confocal microscope, NIS element AR v4.20.03 software (Nikon). Spine density quantification was carried out with Imaris-Microscopy Image Analysis Software by Bitplane (v7.2.3). Myelin basic protein analyses were performed with ImageJ Fiji software (v1.53c). The function Measure of the OrientationJ plugin was used to analyze MBP coherence. Imaging of rabies infected neurons was performed with MacroFluo microscope (Leica), Nis Elements F v3.2 software (Nikon). Behavioral readouts were recorded using the Any-Maze Video Tracking software (v6.34) (Stoelting Co.) connected to a Camera DMK 22AUC03 (Ugo Basile). Electrophysiology data were acquired using a Multiclamp 700B amplifier controlled by pClamp 10 software (Molecular Device), with a Digidata 1322 (Molecular Device). |
| Data analysis | Mouse MRI scans were analyzed with FSL (v6.0), AFNI (v21.0) and ANTS (v2.1), python (v3) and GraphPad (v9.2). Whole brain network modelling was carried out with python (v3). Human MRI scans were analyzed with AFNI (v21.0), MATLAB (R2019a), python (v3) and GraphPad (v9.2). Gene enrichment analysis was carried out with R (v4.1). Spine density, AMPA/NMDA ratio, behavioral scores, viral tracing and MBP were analyzed with GraphPad (v9.2). |

For manuscripts utilizing custom algorithms or software that are central to the research but not yet described in published literature, software must be made available to editors and reviewers. We strongly encourage code deposition in a community repository (e.g. GitHub). See the Nature Research guidelines for submitting code & software for further information.

# Data

Policy information about availability of data

All manuscripts must include a data availability statement. This statement should provide the following information, where applicable:

- Accession codes, unique identifiers, or web links for publicly available datasets
- A list of figures that have associated raw data
- A description of any restrictions on data availability

The authors declare that human MRI and phenotypic data is available in the following public repository (http://fcon_1000.projects.nitrc.org/indi/abide/) and that mouse fMRI scans are available from the corresponding author upon reasonable request. The authors also declare that gene expression data are publicly available in the web portal of the Allen Brain Institute (http://www.brain-map.org/). Source data are provided with this paper.

# Field-specific reporting

Please select the one below that is the best fit for your research. If you are not sure, read the appropriate sections before making your selection.

[x] Life sciences  [ ] Behavioural & social sciences  [ ] Ecological, evolutionary & environmental sciences

For a reference copy of the document with all sections, see nature.com/documents/nr-reporting-summary-flat.pdf

# Life sciences study design

All studies must disclose on these points even when the disclosure is negative.

| | |
|---|---|
| Sample size | Sample size is reported in the figure caption for each experiment. No statistical methods were used to predetermine sample sizes for our experiments, but our sample sizes are comparable or higher to those reported in previous publications on mouse rsfMRI (Liska et al. 2018, Pagani et al. 2019, Bertero et al. 2018, Sutterlin et al. 2018, Sforazzini et al. 2016), dMRI (Liska et al. 2018, Pagani et al. 2019, Sforazzini et al. 2016), behavior (Liska et al. 2018, Pagani et al. 2019), viral tracing (Liska et al. 2018, Pagani et al. 2019, Sforazzini et al. 2016), MBP staining on the same Tsc2 mouse line (Christine Chin-jung Hsieh et al. 2020), AMPA/NMDA ratio (Benthall et al. 2021, Sloley et al. 2021, Duffney et al. 2015, Johansson & Silberberg 2020) and human neuroimaging studies on ASD (Hong et al. 2019, Superkar et al. 2013). |
| Data exclusions | We analyzed scans of participants collected from eight independent laboratories: Kennedy Krieger Institute (KKI), New York University (NYU), Oregon Health and Science University (OHSU), Stanford University (SU), University of California Los Angeles (UCLA, sample 1 and 2), University of Michigan (UM, sample 1) and Yale Child Study Center (YALE), which were the only sites that included children younger than 13 years old with autism and neurotypical controls, with ≥9 individuals per group, and who had full MRI data (i.e., structural and functional). Neurotypical controls did not have any history of mental disorders. No data were excluded for animal studies. |
| Replication | The complex and lengthy design of our mouse studies did not allow us to perform replication studies, as such endeavor would also have important ethical implications in terms of 3R compliance. We however thoroughly randomized genotype and treatment groups, making analysts blind to treatment genotype. This strategy, together with the statistical robustness of our experimental findings, suggest our results would be reproducible across repetition. We finally point out that the observation of spine and behavioral normalization upon rapamycin treatment is per se an independent confirmation of a previously published result (ang et al., 2014). Moreover, to ensure that altered synaptic density in Tsc2 mice described in Tang et al. 2014 could be reliably replicated as well as the rapamycin-mediated rescue, we measured synaptic density in our Tsc2 mice treated with and without rapamycin. All our attempts at replication were successful (Figure 1A and Figure 3A).In the case of human studies, we assessed replicability of our main findings of insular functional hyperconnectivity in autism via a cross-laboratory connectivity analysis we report in Figure 4A.in the form of a conjunction map. In the same panel we similarly quantified the corresponding connectivity strength across all the laboratories. |
| Randomization | For all mouse experiments, animals were chosen based on genotypes. Aged-matched wild-type and mutant littermates were compared to minimize variance in age, genetic background and environment. Mice were then assigned to the group treated with rapamycin or vehicle by using a random number generator as implemented in MATLAB. For human data, individuals were grouped by diagnosis (ASD vs. control population) |
| Blinding | All data acquisition and analysis were performed blind to the genotype/treatment condition. |

# Reporting for specific materials, systems and methods

We require information from authors about some types of materials, experimental systems and methods used in many studies. Here, indicate whether each material, system or method listed is relevant to your study. If you are not sure if a list item applies to your research, read the appropriate section before selecting a response.

## Materials & experimental systems

| n/a | Involved in the study | |
|---|---|---|
| [ ] | [x] | Antibodies |
| [ ] | [x] | Eukaryotic cell lines |
| [x] | [ ] | Palaeontology and archaeology |
| [ ] | [x] | Animals and other organisms |
| [ ] | [x] | Human research participants |
| [x] | [ ] | Clinical data |
| [x] | [ ] | Dual use research of concern |

## Methods

| n/a | Involved in the study | |
|---|---|---|
| [x] | [ ] | ChIP-seq |
| [x] | [ ] | Flow cytometry |
| [ ] | [x] | MRI-based neuroimaging |

# Antibodies

Antibodies used

Primary Antibodies:
Chicken polyclonal anti Green-Fluorescent-Protein (GFP) (AbCam ab13970 1:1000)
Rabbit polyclonal anti Red-Fluorescent-Protein (RFP) (AbCam ab62341 1:500)
Rat monoclonal (clone number 12) anti Myelin Basic protein (MBP) (AbCam ab7349 1:1000)
Secondary Antibodies:
Goat anti Chicken Alexa Fluor 488 secondary antibody Thermofisher (A11039 1:500)
Goat anti Rabbit-HRP secondary antibody Jackson Immunoresearch (111-035-144 1:500)
Donkey anti-Rat IgG (H+L) Alexa Fluor 488 conjugate (ThermoFisher A21208 1:500)

Validation

Antibodies used in this study were validated by the manufacturer who provided references on their websites using the catalog number provided and/or proven to work in the following papers (references below):
- Rabbit polyclonal anti Red-Fluorescent-Protein (RFP) (AbCam ab62341) was already employed in Bertero et al 2018, Liska et al 2018, Pagani et al 2019, Sforazzini et al 2016, following the same experimental protocol used here to label RFP-positive rabies virus-infected neurons.
- Chicken polyclonal anti Green-Fluorescent-Protein (GFP) (AbCam ab13970) was used in many papers as listed by the manufacturer. We also previously used it in Maddaloni et al 2019. In our work the anti GFP antibody was used to stain the AAV-infected neurons with the aim to highlight the dendritic spines.
- Rat monoclonal anti Myelin Basic protein (MBP) (AbCam ab7349) raised against the full-length cow Myelin Basic protein (clone number 12). The antibody was shown in Western blot to bind two MBP isoforms (4 and 14) by the manufacturer. In our lab it had previously shown to successfully image MBP in immunofluorescence data (Liska et al 2018). The same antibody was used by Hsieh et al 2020 in their immunohistochemistry experiment on TSc2 mice.

# Eukaryotic cell lines

Policy information about cell lines

Cell line source(s)

Hek293T cells have been purchased from ATCC (CRL-3216)

Authentication

Cell line was authenticated by the manufacturer. Authentication procedures were not specified by the manufacturer.

Mycoplasma contamination

Cells were tested negative for mycoplasma contamination.

Commonly misidentified lines (See ICLAC register)

No misidentified cell lines were used in this study

# Animals and other organisms

Policy information about studies involving animals; ARRIVE guidelines recommended for reporting animal research

Laboratory animals

Male Tsc2+/- mice (Jackson laboratories, Stock No: 004686) back crossed for more than 10 generations with C57BL/6J mice were used throughout the study. Tsc2+/+ littermates were used as control, "wild-type" mice. Animals were group housed in a 12:12 hour light-dark cycle in individually ventilated cages with access to food and water ad libitum and with temperature maintained at 21 ± 1 ° C and humidity at 60 ± 10%.

Wild animals

No wild animals were used in the study, but only Tsc2+/+ control littermates.

Field-collected samples

This study did not involve field collected samples.

Ethics oversight

Animal studies were conducted in accordance with the Italian Law (DL 26/2014, EU 63/2010, Ministero della Sanità, Roma) and the recommendations in the Guide for the Care and Use of Laboratory Animals of the National Institutes of Health. Animal research protocols were reviewed and consented to by the animal care committee of the Istituto Italiano di Tecnologia and the Italian Ministry of Health. All surgical procedures were performed under anesthesia.

Note that full information on the approval of the study protocol must also be provided in the manuscript.

## Human research participants

Policy information about studies involving human research participants

| | |
|---|---|
| Population characteristics | The population characteristics of the sample are shown in great details in the reference publication https://www.nature.com/articles/mp201378 and summarized in Supplementary Table I |
| Recruitment | All MRI and clinical data has been downloaded from ABIDE, a publicly available dataset (http://fcon_1000.projects.nitrc.org/indi/abide/abide_I.html) |
| Ethics oversight | Ethical approval and informed consent of the ABIDE study was obtained by the local Institutional Review Board of each laboratory, as detailed in (Di Martino et al., 2014). More information is reported in the original work https://www.nature.com/articles/mp201378 |

Note that full information on the approval of the study protocol must also be provided in the manuscript.

## Magnetic resonance imaging

### Experimental design

| | |
|---|---|
| Design type | Resting state fMRI for mice and humans. |
| Design specifications | Resting state fMRI for mice and humans. |
| Behavioral performance measures | NA |

### Acquisition

| | |
|---|---|
| Imaging type(s) | BOLD-functional and diffusion weighted images |
| Field strength | 7T for mice. For humans see the field strength used in each laboratory in the publicly available repository http://fcon_1000.projects.nitrc.org/indi/abide/abide_I.html |
| Sequence & imaging parameters | Mouse rsfMRI: Functional images were acquired with a 7T MRI scanner (Bruker Biospin, Milan – Bruker Paravision v6) and using a 72-mm birdcage transmit coil and a 4-channel solenoid coil for signal reception (Liska et al., 2015). For each session, in-vivo anatomical images were acquired with a fast spin echo sequence (repetition time [TR] = 5500 ms, echo time [TE] = 60 ms, matrix 192 × 192, field of view 2 × 2 cm, 24 coronal slices, slice thickness 500 µm). Co-centered single-shot BOLD rsfMRI time series were acquired using an echo planar imaging (EPI) sequence with the following parameters: TR/TE = 1000/15 ms, flip angle 30°, matrix 100 × 100, field of view 2.3 × 2.3 cm, 18 coronal slices, slice thickness 600 µm for 1920 volumes.<br><br>Mouse dMRI: High-resolution DW images morpho-anatomical T2-weighted MR imaging of mouse brains was performed using a 72 mm birdcage transmit coil, a custom-built saddle-shaped solenoid coil for signal reception. Each DW data set was composed of 8 non-DW images and 81 different diffusion gradient-encoding directions with b = 3000 s/mm2 (∂ = 6 ms, Δ = 13 ms) acquired using an EPI sequence with the following parameters: TR/TE = 13500/27.6 ms, field of view 1.68 × 1.54 cm, matrix 120 × 110, in-plane spatial resolution 140 × 140 µm, 54 coronal slices, slice thickness 280 µm, number of averages 20 as recently described (Pagani et al., 2019).<br><br>Human rsfMRI: For details of sequence and scan parameters for each collection site see the publicly available repository http://fcon_1000.projects.nitrc.org/indi/abide/abide_I.html |
| Area of acquisition | Whole brain imaging for both mice and humans |

Diffusion MRI ☒ Used ☐ Not used

Parameters: Mouse dMRI: 81 different diffusion gradient-encoding directions with b = 3000 s/mm2, single shell, no cardiac gating

### Preprocessing

| | |
|---|---|
| Preprocessing software | Mouse rsfMRI: removal of first 50 volumes (AFNI v.21 3dTcat), despiking (AFNI 3dDespike), motion correction (FSL v.6 mcflirt), skull-stripping (FSL bet), spatial normalization with affine and diffeomorphic registration (ANTS v.2 antsRegistration + antsApplyTransforms) to a skull-stripped reference BOLD template, calculation of motion traces of head realignment parameters (3 translations + 3 rotations) and mean ventricular signal (corresponding to the averaged BOLD signal within a reference ventricular mask, FSL, fslmeants), regression of nuisance parameters (FSL, fsl_regfilt), band-pass filtering (AFNI 3dBandpass) and spatial smoothing (AFNI, 3dBlurInMask).<br><br>Mouse dMRI: Correction for eddy current distortions (FSL eddy_correct), skull-stripping (FSL bet), manual correction of brainmask using ITK-SNAP (Yushkevich et al., 2006).<br><br>Human rsfMRI: AFNI: Slice acquisition correction, rigid-body head movement correction, obliquity transform to the structural image, affine co-registration to the skull-stripped structural image, nonlinear warping to MNI space (MNI152 template), spatial smoothing, confound signal regression, bandpass filtering. MATLAB Brain Wavelet toolbox: wavelet denoising. ANTsR: |

CompCorr.

Normalization

Mouse rsfMRI and dMRI: data was registered with a combination of affine and non-linear transformations.

Human rsfMRI: data underwent affine co-registration to structural image and then nonlinear warping to MNI space

Normalization template

Mouse rsfMRI: consensus average BOLD template

Mouse dMRI: consensus average FA template

Human rsMRI: MNI space

Noise and artifact removal

Mouse rsfMRI: Nuisance variables of 6 head realignment parameters and mean ventricular signal. Band-pass filtering 0.01-0.1Hz.

Mouse dMRI: Correction for eddy current distortions.

Human rsfMRI: Nuisance variables of head motion and mean ventricular signal (13 parameters). Band-pass filtering 0.01-0.1 Hz. Confirmatory analysis was carried out with mean white matter signal and CompCorr of white matter signal.

Volume censoring

Human rsfMRI: Confirmatory analysis was carried out with volume censoring of mean FD<0.2 mm

## Statistical modeling & inference

Model type and settings

Mouse rsfMRI: multivariate analyses including genotype and treatment as factor

Mouse dMRI: multivariate analyses including genotype as factor

Human rsfMRI: Multivariate analyses controlled for age, sex, IQ, site, number of volumes in addition to including the group factor.

Effect(s) tested

Mouse rsfMRI: Pearson's correlation, one-way ANOVA with post-hoc unpaired t-tests FDR-corrected

Mouse dMRI: multiple unpaired t-tests FDR-corrected

Human rsfMRI: Pearson's correlation, multiple unpaired t-tests FDR-corrected, Cohen's d for effect size

Specify type of analysis: ☐ Whole brain ☐ ROI-based ☒ Both

Anatomical location(s)

Mouse rsfMRI: Based on intergroup differences of spatially unbiased global connectivity mapping

Mouse dMRI: Based on intergroup differences of FA

Human rsfMRI: Based on intergroup differences of spatially unbiased global connectivity mapping

Statistic type for inference
(See Eklund et al. 2016)

Clusterwise

Correction

Voxelwise threshold: (|t| > 2, p < 0.05) and family-wise error (FWER) cluster-corrected using a cluster threshold of p < 0.01

## Models & analysis

| n/a | Involved in the study |
| --- | --- |
| ☐ | ☒ Functional and/or effective connectivity |
| ☐ | ☒ Graph analysis |
| ☐ | ☐ Multivariate modeling or predictive analysis |

Functional and/or effective connectivity

Pearson's correlation

Graph analysis

Unbinarised global connectivity

Multivariate modeling and predictive analysis

Mouse rsfMRI: genotype and treatment

Mouse dMRI: genotype

Human rsfMRI: diagnosis (ASD vs. CTR)
